# *M^2^ara*: unraveling metabolomic drug responses in whole-cell MALDI mass spectrometry bioassays

**DOI:** 10.1093/bioinformatics/btae694

**Published:** 2024-11-18

**Authors:** Thomas Enzlein, Alexander Geisel, Carsten Hopf, Stefan Schmidt

**Affiliations:** Center for Mass Spectrometry and Optical Spectroscopy (CeMOS), Mannheim University of Applied Sciences, Mannheim 68165, Germany; Center for Mass Spectrometry and Optical Spectroscopy (CeMOS), Mannheim University of Applied Sciences, Mannheim 68165, Germany; Center for Mass Spectrometry and Optical Spectroscopy (CeMOS), Mannheim University of Applied Sciences, Mannheim 68165, Germany; Medical Faculty, Heidelberg University, Heidelberg 69117, Germany; Mannheim Center for Translational Neuroscience (MCTN), Medical Faculty Mannheim, Heidelberg University, Mannheim 68167, Germany; Center for Mass Spectrometry and Optical Spectroscopy (CeMOS), Mannheim University of Applied Sciences, Mannheim 68165, Germany

## Abstract

**Summary:**

Fast computational evaluation and classification of concentration responses for hundreds of metabolites represented by their mass-to-charge (*m/z*) ratios is indispensable for unraveling complex metabolomic drug actions in label-free, whole-cell Matrix-Assisted Laser Desorption/Ionization Mass Spectrometry (MALDI MS) bioassays. In particular, the identification of novel pharmacodynamic biomarkers to determine target engagement, potency, and potential polypharmacology of drug-like compounds in high-throughput applications requires robust data interpretation pipelines. Given the large number of mass features in cell-based MALDI MS bioassays, reliable identification of true biological response patterns and their differentiation from any measurement artefacts that may be present is critical. To facilitate the exploration of metabolomic responses in complex MALDI MS datasets, we present a novel software tool, *M^2^ara*. Implemented as a user-friendly R-based shiny application, it enables rapid evaluation of Molecular High Content Screening (MHCS) assay data. Furthermore, we introduce the concept of Curve Response Score (CRS) and CRS fingerprints to enable rapid visual inspection and ranking of mass features. In addition, these CRS fingerprints allow direct comparison of cellular effects among different compounds. Beyond cellular assays, our computational framework can also be applied to MALDI MS-based (cell-free) biochemical assays in general.

**Availability and implementation:**

The software tool, code, and examples are available at https://github.com/CeMOS-Mannheim/M2ara and https://dx.doi.org/10.6084/m9.figshare.25736541.

## 1 Introduction

Whole-cell MALDI MS assays are a versatile Molecular High Content Screening (MHCS) technology that unravels the metabolomic complexity of drug responses in a biological system (Weigt *et al.* 2018, 2019, Unger *et al.* 2020, 2021, Dueñas *et al.* 2023). In contrast to conventional HCS technologies that focus primarily on morphometric or functional measures such as cell size, neurite length, dendrite branching, or calcium signaling (Yeyeodu *et al.* 2010, Wu *et al.* 2019), MHCS refers to the fact that MS cell assays can monitor hundreds of metabolites simultaneously, providing a snapshot of the metabolome. This enables targeted, i.e. known target molecules, as well as untargeted analysis, i.e. identification of potential cellular response markers or the discovery of phenotypic changes upon treatment. However, to date, the interpretation and classification of hyperspectral MS assay data remains a computational challenge in bioanalytics, pharmacology as well as chemical biology and life science research. In particular, in phenotypic assays where the mode of action of drugs can be investigated in a target-agnostic manner, sophisticated data interpretation pipelines are essential to transform individual cellular *metabolomic fingerprints* into a meaningful and simple form that can be classified, sorted, and visualized based on well-defined scores. Hereby, mass-specific concentration response curves are deduced, followed by their classification and characterization using given assay metrics. Understanding metabolomic responses can enable the identification of useful pharmacodynamic biomarkers and thus allows the determination of potencies and effect sizes of test compounds in early drug discovery including early drug safety testing. Nevertheless, there is no common open-source or proprietary software tool capable of performing concentration–response regression analysis in an easy-to-use fashion of complex MALDI MS data. Moreover, implementations designed for the classification and scoring of cellular metabolomic fingerprints across compound libraries or cell lines are missing (Di Veroli *et al.* 2015, Ritz *et al.* 2015, Wang *et al.* 2020, Unger *et al.* 2021). Recently, a method has been introduced to address the issue of statistical significance in concentration–response curve analysis, allowing concentration–response relationships for e.g. large proteomics datasets to be visualized, evaluated, and understood (Bayer *et al.* 2023).

To facilitate the exploration of cellular drug effects in comprehensive MALDI MS metabolomic datasets and to link measured effects to multiple drug-targets in high-throughput screens, we introduce a novel software tool, *M^2^ara*, for the interpretation of whole-cell MALDI MS bioassays, which includes a scoring system to filter for significant mass features. It is implemented as an open-source R-based *shiny* application, including several modifications and additions compared to our previously published MALDIcellassay R package (Unger *et al.* 2021). Specifically, we generated a *shiny* based graphical user interface, including insightful plots to visualize spectra, dose-response curves, assay metrics, and experimental parameters, introduced metrics for assay quality, and added functionalities for outlier detection, clustering of curves with similar shape as well as the option to perform sparse principal component analysis (PCA) as a multivariate alternative to univariate dose-response curves. In addition, *M^2^ara* allows to import data in the Bruker Flex (*.fid) format as well as the open mzML standard. All modifications are included in the latest version of the MALDIcellassay R package and are available in the Comprehensive R Archive Network (CRAN).

## 2 Results

**Figure 1.**
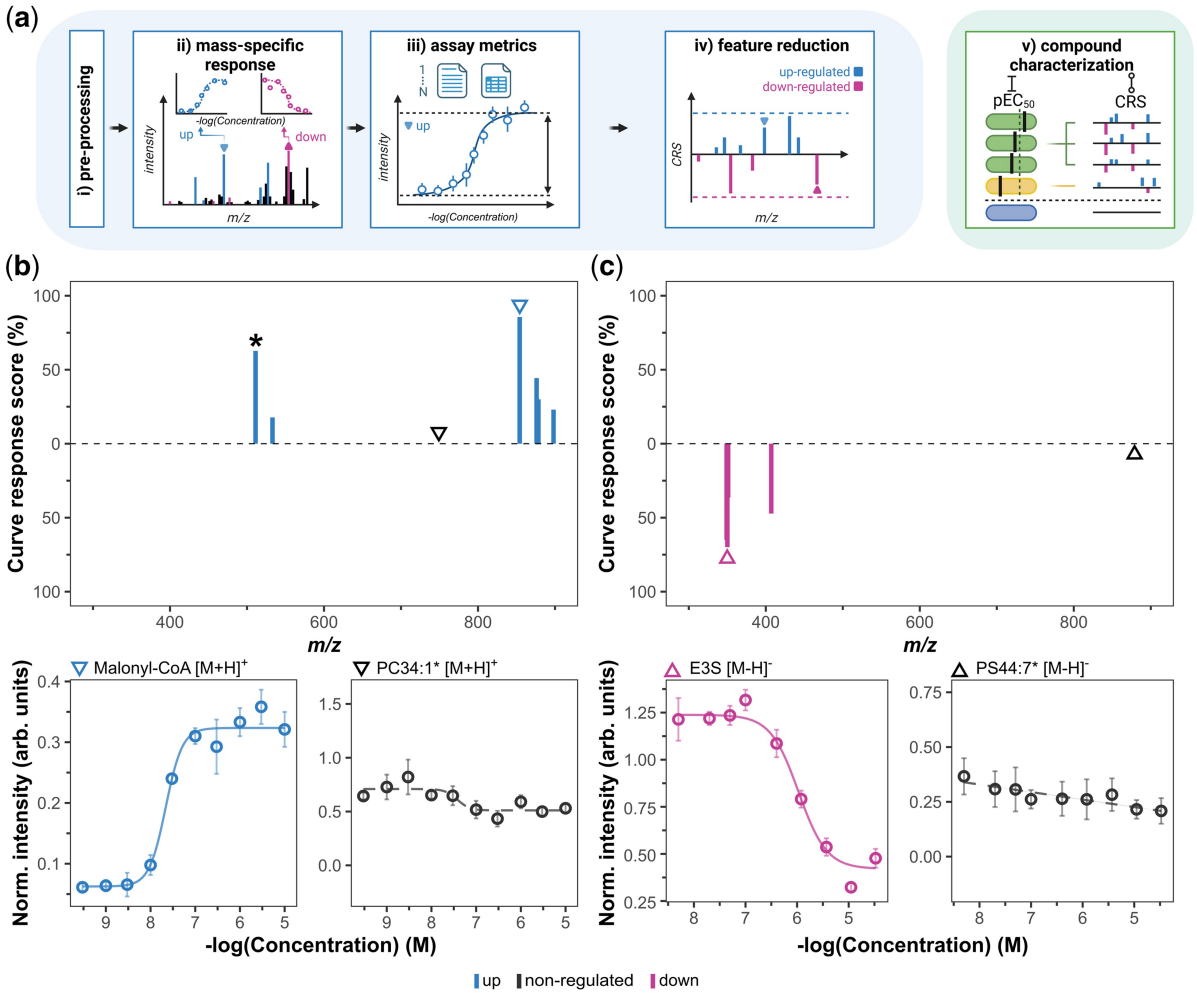
Schematic of the computational framework of M2ara for the interpretation and classification of metabolic drug responses in complex MALDI-TOF-MS assays. (a) The data interpretation pipeline of *M^2^ara* includes (i) data-preprocessing, (ii) fitting of a sigmoidal response curve, (iii) calculation of assay quality measures, and (iv) feature reduction based on an introduced Curve Response Score, a composite score taking into account different assay metrics. The drug action can be repeatedly calculated for multiple compounds (v), allowing for compound classification based on their potency or direct comparison of metabolic response patterns using CRS fingerprints outside of *M^2^ara*. Created in BioRender. https://BioRender.com/u34m733. (b) CRS fingerprint for the FASN inhibitor MALDI cell assay presented in Weigt *et al.* (2019), for A549 cells treated with compound GSK2194069. Lower panel shows characteristic drug–response curves of two selected mass features, malonyl-CoA (left) (*m/z* 854.1, [M + H]^+^) as an up-regulated responder as well as a nonresponder (PC34:1, putative, *m/z* 760.6, right). *Asterisk marks CRS of *m/z* 511.1, [M + H]^+^, CDP-choline, another reported mass feature from Weigt *et al.* (2019) (see Supplementary Fig. S3). (c) CRS fingerprint for the OATP2B1 inhibitor erlotinib based on a cell assay presented in Unger *et al.* (2020). Lower panel shows characteristic drug–response curves of two selected mass features E3S (estrone 3-sulfate) (*m/z* 349.1, [M−H]^−^) as a down-regulated responder, and *m/z* 888.6 (*PS44:7 [M−H]^−^, putative) as a nonregulated. Uncertainties are presented as standard deviation of four technical replicates.

The computational pipeline of *M^2^ara* (Fig. 1a, Supplementary Fig. S1) is designed to decipher the molecular complexity of drug responses and to provide response characteristics in cellular systems. To this end, raw MS data (Bruker raw data or mzML) is first imported using the *MALDIquantForeign* R-package (Gibb and Strimmer 2012) followed by (user-defined) data pre-processing such as baseline removal using the TopHat method, Savitzky-Golay smoothing of the spectra, square-root transformation to stabilize variance of the data and filtering for monoisotopic peaks (Fig. 1a, i). Next, *M^2^ara* offers recalibration of the data to a selected peak, e.g. using a *m/z* value of an internal standard, normalization (total ion current, internal standard, etc.) and alignment of the spectra. Since the MALDI target plate layout commonly includes several technical replicates per condition, potential outliers are highlighted in the *plate map* using Chauvenet’s Criterion (Chauvenet 1871) as well as information on the total ion current or signal intensity for a given feature per spot. After pre-processing, the mean spectra for all concentrations are calculated and a four-parametric logistic regression curve is fitted for each *m/z* feature (Fig. 1a, ii).

Subsequently, the curve characteristics, i.e. the effect size and the effect potency, are derived using a four-parameter log-logistic regression model, as detailed in the Supplementary Information. In this model, the effect potency is determined by the negative logarithmic value of the half-maximal effective concentration pEC50. The magnitude (ratio between upper and lower asymptotes) and the sign of the slope, both of which are directly deduced from the fit, provide the effect size. In order to assess the variability within the assay data relative to the effective window size determined by the end-points for high- and low-concentrations and to obtain the root-mean-square deviation of the response data relative to the log-logistic model fit, two factors, $F_{Z}$ and $F_{V}$, respectively, were introduced (see Supplementary Information and Supplementary Fig. S3). Both factors are based on the original definitions of assay quality measures, as proposed by Zhang *et al.* and Ravkin *et al.*, and are applied for the judgement of the curve-response data (Fig. 1a, iii). In addition, we implemented a Curve Response Score (CRS) to yield a dedicated feature reduction and thus a focused selection and ranking of significant mass features (Fig. 1a, iv). The corresponding CRS fingerprint provides a unique visualization of regulated mass features unraveling metabolic responses essential for exploring cellular metabolic alterations for a given compound or even across different compounds. The CRS is a combination of the aforementioned factors ($F_{Z}$ and $F_{V}$) and the magnitude of the curve (log_2_FC), with all three variables weighted equally. The intention behind this definition is to create a dedicated list of regulated and nonregulated features. It offers a relative ranking of all regulated features within one dataset and allows the user to make an initial judgement of their data in terms of assay quality. Based on the given shape, e.g. slope, and results of the used score, response curves are classified as either up- or down-regulated or even nonregulated. Results are presented to the user in a convenient table format and plots, including response curves as well as MS spectra. All performance measures can be exported as a .csv file for further analysis.

We introduce and benchmark *M^2^ara* by presenting previously published data (Supplementary Table S1), ranging from a cell-based MALDI MS fatty acid synthase (FASN) inhibitor assay (Weigt *et al.* 2019), a MALDI MS cellular screening assay for compounds that block the uptake of the substrate estrone-3-sulfate via the OATP2B1 transporter (Unger *et al.* 2020), and a phenotypic, i.e. target-agnostic cell-based assay for tyrosine-kinase inhibitors (Weigt *et al.* 2018) to a complement dependent cytotoxicity (CDC) assay for potency assignment of therapeutic antibodies (Schmidt *et al.* 2024). For the purposes of comparison, the pre-processed assay data from Weigt *et al.* (2019), Unger *et al.* (2020), and Schmidt *et al.* (2024) were processed using CurveCurator (Bayer *et al.* 2023).

The direct comparison yielded similar results (see Supplementary Figs S3–S5), with the method proposed by Bayer *et al.* (2023) being more inclusive. *M^2^ara* was developed with cell-based assays in mind, yet it can be utilized for any experiment involving treatments at different concentrations and MALDI MS as a means of detection. Hence, beyond cellular assays, our computational framework can be used for MALDI MS-based biochemical assays in general. Examples of a previously published γ-secretase in vitro inhibition assay (Koch *et al.* 2023) (Supplementary Fig. S6) and cell-free caspase-6 inhibitor assay (Supplementary Fig. S6, https://www.ppscreeningcentre.com/label-free-mass-spectrometry-ultra-highthroughput-screening/) are provided.

Overall, results for each case study re-evaluated with *M^2^ara* are summarized in Supplementary Table S2 including the number of *m/z* features that are either up- or down-regulated (CRS > 0). Moreover, the values for the quality scores of the *m/z* feature with the highest CRS are also presented for each case. Exemplarily and in agreement with Weigt *et al.* (2019) the CRS fingerprint of the FASN bioassay (Fig. 1b) features a CRS for the up-regulated metabolite malonyl-CoA (*m/z* 854.1, [M + H]^+^, annotation taken from original publication) of the fatty acid synthesis pathway of 94.0%, whose corresponding response curve is depicted in Fig. 1b. Furthermore, the *m/z* feature with the second-highest CRS is reported to be the downstream metabolite cytidine diphosphate (CDP)-choline of the phospholipid biosynthesis (Supplementary Fig. S8). In addition, a nonregulated mass feature (*m/z* 760.6, likely PC34:1 [M + H]^+^) with a CRS of 0% is presented for comparison. Assessment of the CRS fingerprints for the three technical replicates reported in Weigt *et al.* (2019: 20) yield similar patterns (Supplementary Fig. S9). A direct comparison of the CRS fingerprint against each individual metric (Supplementary Fig. S10) demonstrates the ability of the CRS fingerprint to serve as a rapid scoring system for the identification of up- or down-regulated *m/z* features, thereby emphasizing the importance of combining different scoring metrics.

A similar simple CRS fingerprint was computed for the OATP2B1 transporter inhibition assay (Fig. 1c and Supplementary Fig. S11 for three technical replicates). As expected, the *m/z* value for the down-regulated substrate estrone-3-sulfate (*m/z* 349.1, [M−H]^−^, annotation taken from original publication) yielded the highest CRS of 75.3%. More complex patterns were found for the phenotypic assays presented by Weigt *et al.* (2018) (Supplementary Fig. S12) and Schmidt *et al.* (2024), with e.g. glutathione (GSH) as the most significant response marker identified in the latter case study (Supplementary Fig. S13). For the latter, we have computed the CRS for six different conditions, where in each iteration the data point for the lowest intensity is removed from the dataset (Supplementary Fig. S14) to evaluate the case, where end-points are not covered by the experimentally chosen concentration range. As indicated, even for the condition, where only half of the response curve is covered (Supplementary Fig. S11d), GSH shows up in the CRS fingerprint with a CRS of 68.4%. Experimentally, if such a behavior would be recognized during assay development, the concentration range would be expanded to cover the full range of the drug-effect.

Expanding the evaluation to multiple drugs allows compound classification based on either a single mass feature or the entire CRS fingerprint (Fig. 1a, v), exemplified for a small number of compounds using the FASN inhibitor dataset (Supplementary Fig. S15a and c). Supplementary Figure S15a shows the pEC50 as well as the CRS for different FASN inhibitors using malonyl-CoA (*m/z* 854.1, [M+H]^+^) enrichment as the read-out. Supplementary Figure S15b on the other hand shows all *m/z* features with a nonzero CRS versus their respective pEC50 values for different drugs and is an example of an untargeted approach where the response fingerprint is of interest. This can be useful to identify off-target-effects of known compounds.

Besides univariate analysis, *M^2^ara* offers a set of multivariate analysis tools. For example, the global response can be visualized with a PCA (Supplementary Fig. S15c). This approach can serve as a feasibility check for experiments at an early stage of assay development. Moreover, by leveraging a sparse PCA that uses an L1-norm to represent the large feature space with just a small subset of *m/z* features, it is also possible to identify multivariate responses, where all curves examined alone might not cross the threshold to be identified as valid responses, but taken together provides insight into complex mechanisms.

Finally, to assess concentration response similarities, *M^2^ara* features the classification of response curves based on their sigmoidal shape (Supplementary Fig. S6c). This serves as an additional selection or similarity feature for deciphering metabolic responses. In particular, if response markers with a positive CRS are present, it may help to identify other *m/z* features with the same (but weaker/noisier) response curve characteristics. An example would be the identification of additional dysregulated Aβ-species that, although below the CRS quality threshold, still follow the same trend as the Aβ-species detected by CRS alone (Supplementary Fig. S6c and d). Additionally, this technique might also be used to identify markers with a reverse response.

## 3 Discussion

In the last decade, mass spectrometry based assay technology has emerged as a promising high-throughput technology in drug discovery (Unger *et al.* 2021, Dueñas *et al.* 2023) including MALDI MS or its variants like IR-MALDESI MS or acoustic mist ionization/acoustic ejection MS (Belov *et al.* 2020, Simon *et al.* 2020, 2021, Pu *et al.* 2023). Standardized protocols exist for the establishment of both, biochemical or cellular assays (De Cesare *et al.* 2020, Unger *et al.* 2020). The latter provide a unique framework for early drug discovery as compounds are directly tested in cells, offering the potential for identification of more specific target compounds (Unger *et al.* 2020, Pu *et al.* 2023). Beyond that, for phenotypic cellular experiments, in which the metabolic response to a pharmaceutical reagent is usually unknown, computational data evaluation pipelines implemented in a single platform are indispensable to handle data load, data complexity and to provide a fast and user-friendly way for data visualization and data interpretation. With *M^2^ara* it becomes straightforward to perform response curve analysis for the identification of e.g. potential response markers as well as mode-of-action studies. *M^2^ara* facilitates the evaluation and classification of mass-specific response curves enabling fast decision making in assay development. For compound library screens, *M^2^ara* provides statistical tools for the visualization and analysis of screening results including similarity tests and potency assignments of compounds. For next level analytical assay analysis, the import of trapped ion mobility data could be included in future (Willems *et al.* 2021, Luu *et al.* 2022). Furthermore, advanced machine learning models might also help to further improve statistical evaluation and classification of response curves, feature classification and pattern recognition.

In summary, we present a novel, open-source software tool for the evaluation and interpretation of MHCS MALDI MS datasets in whole-cell phenotypic and mechanistic cell assay as well as enzymatic assays. The data analysis pipeline facilitates robust feature identification in minutes by introducing a Curve Response Score. Those drug-dependent metabolic fingerprints are reduced in features space to allow for fast and significant similarity tests across compounds. Novel response *m/z* biomarkers can be identified rapidly by ranking of significant features including potency assignment and unequivocally identified on the MS2 level in follow-up experiments. *M^2^ara* can be used for both targeted and untargeted analysis of concentration-dependent responses. In the absence of a definitive understanding of the mode of action, the discovery of potential markers in *M^2^ara* represents an initial but indispensable step in the rigorous characterization and identification (e.g. with MS2 analysis) of such a marker.

Predominantly, *M^2^ara* serves as a computational tool in whole-cell MALDI MS assay development for biomarker identification, hit confirmation and follow up characterization in high-throughput screens and the investigation of alterations in cell metabolism upon drug treatment.

## Supplementary Material

btae694_Supplementary_Data

## Data Availability

The data underlying this article (Weigt *et al.* 2018, 2019, Unger *et al.* 2020, Koch *et al.* 2023, Schmidt *et al.* 2024) are available in FigShare, at https://doi.org/10.6084/m9.figshare.25736541.v2. The Cas-6 data were provided by Pivot Park Screening Centre by permission. *Data will be shared on request to the corresponding author with permission of* Pivot Park Screening Centre.
