# Supplementary material for: M2ara: unraveling metabolomic drug responses in whole-cell MALDI mass spectrometry bioassays
Source: Bioinformatics. 2024 Nov 18;40(11):btae694. doi: 10.1093/bioinformatics/btae694 (PMC11601156; doi:10.1093/bioinformatics/btae694)
Supplement: btae694_Supplementary_Data [file btae694_supplementary_data.docx]

*M^2^ara*: unraveling metabolomic drug responses in whole-cell MALDI mass spectrometry bioassays

Thomas Enzlein^1^, Alexander Geisel^1, †^ and Carsten Hopf^1, 2, 3, *^ Stefan Schmidt^1, *^

^1^Center for Mass Spectrometry and Optical Spectroscopy (CeMOS), Mannheim University of Applied Sciences, Paul-Wittsack-Str. 10, 68165 Mannheim

^2^Medical Faculty, Heidelberg University, Im Neuenheimer Feld 280, 69117 Heidelberg, Germany

^3^Mannheim Center for Translational Neuroscience (MCTN), Medical Faculty Mannheim, Heidelberg University, Theodor Kutzer-Ufer 1-3, 68167 Mannheim, Germany

^*^To whom correspondence should be addressed.

# Supplementary information

**Assay quality parameters and ranking of mass features in *M^2^ara***

Dose-response curves are fitted using a 4-parameter log-logistic (4PL) regression model, implemented in the R-package *nplr* (Commo and Bot 2016). The regression model used to describe the shape of the concentration-effect curves is known as the Hill equation (Giraldo *et al.* 2002) and is expressed in the following form:

| $f\left( c \right)=a_{l}+\frac{a_{u}-a_{l}}{\left( 1+{10}^{(h\cdot(c_{1}-c))} \right)}$, | (1) |
| --- | --- |

where $a_{u}$ and $a_{l}$ are the upper and lower asymptotes, respectively, $h$ denotes the Hill coefficient and $c_{1}$ the point of inflection, i.e. the point of the S-shaped curve halfway between $a_{u}$ and $a_{l}$. The 4PL regression model is governed by the effect potency, denoted as the logarithmic value of the half maximal effective concentration ${\mathrm{pEC}_{50}=c}_{1}$, the effect size, i.e. the magnitude, expressed as the log_2_FC (fold-change),

| $\log_{2}\mathrm{FC}= \log_{2} \frac{a_{u}}{a_{l}}$, | (2) |
| --- | --- |

and the slope. The slope is negative for a down regulated mass feature and positive for an up regulated mass feature of the sigmoidal curve shape. In addition, $\left| a_{u}-a_{l} \right|$ denotes the window size of the sigmoidal curve.

In pharmaceutical industry and research, the quality of a bioassay is assessed by common metrics that rely on a negative and positive control (Zhang, Chung and Oldenburg 1999; Ravkin *et al.* 2004; Iversen *et al.* 2006). However, in order to be able to explore unknown cellular drug effects in whole-cell MALDI MS bioassays and to classify *m/z* features as either up-, down- or even non-regulated, characteristic measures need to be deduced from the concentration response data directly. In light of the findings presented in Zhang et al, within *M^2^ara* we propose the introduction of a factor $F_{Z}$ for the assessment of the variability within the assay data relative to the effective window size defined by

| $F_{Z}:=1- \frac{3\times\left( \sigma_{l}+\sigma_{u} \right)}{\left\vert\mu_{u}-\mu_{l} \right\vert}.$ | (3) |
| --- | --- |

Hereby, ${(\mu}_{u}, \sigma_{u}$) and ${(\mu}_{l}, \sigma_{l}$), denotes the mean and its uncertainty of the two outmost data points on either side of the dose-curve, i.e. those data points for the highest and lowest drug concentrations used experimentally. Usually, but not necessarily, those data points present the end-points of the drug-effect. In addition, by following the work of (Bray and Carpenter 2004; Zhang 2007) for the definition of the Strictly Standardized Mean Difference (SSMD), a similar factor $F_{S}$ is implemented with

| $F_{S}:= \frac{\left\vert\mu_{u}-\mu_{l} \right\vert}{\sqrt{\left( \sigma_{u}^{2}+ \sigma_{l}^{2} \right)}}.$ | (4) |
| --- | --- |

In addition, based on the definition of the V-factor in (Ravkin *et al.* 2004) another factor $F_{V}$ is introduced to assess the root-mean-square deviation of the response data relative to the log-logistic model fit, determined by

| $F_{V}:=1-6\cdot\frac{\sigma_{f}}{\left\vert a_{u}-a_{l} \right\vert}$, | (5) |
| --- | --- |

with

| $\sigma_{f}=\sqrt{\frac{1}{N}\sum\left( f_{\exp}-f \right)^{2}}$, | (6) |
| --- | --- |

where $\sigma_{f}$ is the standard deviation of the residuals of the 4-parameter non-linear regression model *f*, calculated from the experimental (exp) data and the model. Hereby, Eq. (5) reflects the goodness of the fit and thus the variance within all data points described by the 4PL model. Third, the effect size is calculated according to Eq. (3).

*M^2^ara* enabling quick feature identification and thus decision making by the implementation of a simple, one-dimensional score, the *Curve Response Score (CRS)*, and its visualization, the *CRS fingerprint*, for robust ranking of mass features as potentially potent concentration-response markers. The CRS is based on the aforementioned factors to account for the effect size of the response curve (log_2_FC), the variability within the assay data relative to the effective window size determined by the end-points for high and low drug concentrations ($F_{Z}$) and the root-mean-square deviation of the response data relative to the log-logistic model fit ($F_{V}$). As default, it is defined by

| $\mathrm{CRS}:=\left\{ \begin{matrix} 0 & \mathrm{if}F_{Z}<-0.5\mathrm{or}F_{V}<-0.5 \\ \left( fcScore+vScore+zScore \right)/3 & \text{otherwise } \end{matrix} \right..$ | (7) |
| --- | --- |

Hereby,

| $fcScore:= \left\{ \begin{matrix} 1 & \mathrm{if}\left\vert\text{log}_{2}\mathrm{FC} \right\vert> {\text{log}_{2}\mathrm{FC}}_{\max} \\ \left\vert\text{log}_{2}\mathrm{FC} \right\vert/{\text{log}_{\text{2}}\text{FC}}_{\text{max}} & \text{otherwise } \end{matrix} \right.$, | (8) |
| --- | --- |

$\mathrm{with}\log_{\text{2}}FC_{\max}=2.59$,

| $vScore:= F_{V}$ | (9) |
| --- | --- |

and

| $zScore:= \left\{ \begin{matrix} 1 & \text{if} F_{Z}\geq0.5 \\ Z_{\mathrm{mod}}^{'}/0.5 & \text{if} 0.5>F_{V}>-0.5 \\ 0 & \text{otherwise } \end{matrix} \right.$. | (10) |
| --- | --- |

According to Eq. (6), the CRS combines three measures weighted equally that are used to describe the quality of a response curve: the effect size defined by Eq. (2) and incorporated in the fcScore, the $F_{V}$ factor being equal to the vScore and the $F_{Z}$ factor used in the definition of the zScore. In the fcScore, the log_2_FC is normalized by and thresholded at ${\text{log}_{2}\mathrm{FC}}_{\max}=2.59.$ The factor is chosen to not overrate features that exhibit substantial changes. The restriction of the $F_{Z}$ factor within the zScore is made due to the common interpretation of the *Z*’ factor (Zhang, Chung and Oldenburg 1999). For $F_{Z}>0.5$a bioassay is said to be excellent, since for $\sigma_{l}=\sigma_{u}$ a value of 0.5 is equivalent to a separation of 12 standard deviations between $\mu_{u}$ and $\mu_{l}$. Accordingly, a value of -0.5 is equivalent to a separation of 3 standard deviations between $\mu_{u}$ and $\mu_{l}$ for $\sigma_{l}=\sigma_{u}$. The rather moderate lower cutoff is in particular of importance for assay development and optimization, allowing still for a relatively high variance in the data. Another example is presented in **Fig. S6,** where the response for the Aβ1-42 molecule yield a $F_{Z}$ of -0.23, a $F_{V}$of 0.5 and a log_2_FC of -1.65 and therefor a CRS of 22.4 %. In contrast, the value of the CRS for Aβ1-42 % is about a factor of three higher ($CRS=58.0$), with $F_{Z}=0.16$ , $F_{V}=0.41$ and log_2_FC = -2.76, respectively.

**Table S1: Overview of the MALDI MS case studies used in this work.** ^1^Compounds for which assay data was analysed within this work. ^2^Overexpressing OATP2B1. ^3^Identities (IDs) taken from original publication. Abbreviations: CDC: complement dependent cytotoxicity; GSH: Glutathione, E3S: estrone-3-sulfate.

| **Description** | **Re-cal. *m/z*** | **Normalization method** | **Type** | **Cell-based** | **Cell line** | **Compounds^1^** | **Reported  *m/z* features** | **IDs^3^** | **Reference** |
| --- | --- | --- | --- | --- | --- | --- | --- | --- | --- |
| FASN inhibitor assay | 857.13 | m/z | Mechanistic | yes | A549 | GSK2194069  GSK2592200A  GSK2647828A  GSK2889978A  GSK837149A | *m/z* 854.1, [M+H]^+^ | malonyl-CoA | Weigt et al. 2019 |
|  |  |  |  |  |  |  | *m/z* 511.1, [M+H]^+^ | CDP-choline |  |
| OATP2B1 transporter assay | 354.14 | m/z | Mechanistic | yes | HEK293**^2^** | Erlotinib | *m/z* 349.1, [M-H]^-^ | E3S | Unger et al. 2020 |
| caspase-6 inhibitor assay | 631.29 | m/z | Mechanistic | no |  | Ivachtin | *m/z* 625.3, [M+H]^+^ | QVEID | White paper of PPSC |
| γ-secretase inhibition assay | 3263.5 | TIC | Mechanistic | no |  | DAPT | *m/z* 4074, [M+H]^+^ | Aβ1-37 | Koch et al. 2023 |
|  |  |  |  |  |  |  | *m/z* 4131, [M+H]^+^ | Aβ1-38 |  |
|  |  |  |  |  |  |  | *m/z* 4327, [M+H]^+^ | Aβ1-40 |  |
|  |  |  |  |  |  |  | *m/z* 4514, [M+H]^+^ | Aβ1-42 |  |
|  |  |  |  |  |  |  | *m/z* 4615, [M+H]^+^ | Aβ1-43 |  |
|  |  |  |  |  |  |  | *m/z* 4927, [M+H]^+^ | Aβ1-45 |  |
| tyrosine-kinase inhibitor assay | 760.59 | TIC | Phenotypic | yes | K562 | Dasatinib | *m/z* 826.6, [M+H]^+^ | PC 36:1 K | Weigt et al. 2018 |
|  |  |  |  |  |  |  | *m/z* 616.2, [M]^+^ | Heme B |  |
| CDC assay | None | TIC | Phenotypic | yes | Raji | Rituximab | *m/z* 306.1, [M-H]^-^ | GSH | Schmidt et al. 2024 |

**Table S2: Summarized results from benchmark datasets.** ^1^Peaks detected from average spectra after pre-processing. ^2^Selecting the monoisotopic peak from the isotopic envelope. Molecular identity (ID) taken from original publication.

| **Description** | **Ion mode** | **Number of peaks in dataset^1^** | **Up/down regulated peaks with CRS > 0** | **m/z feature with highest CRS / molecular ID^2^** | **CRS (%)** | $F_{Z}$ | $F_{V}$ | **log_2_FC** |
| --- | --- | --- | --- | --- | --- | --- | --- | --- |
| FASN inhibitor assay | positive | 252 | 8 | *m/z* 854.1, [M+H]^+^  malonyl-CoA | *94.0* | *0.7* | *0.8* | *3.5* |
|  |  |  | 0 |  |  |  |  |  |
| OATP2B1 transporter assay | negative | 302 | 0 | *m/z* 349.1, [M-H]^-^  E3S | *75.3* | *0.3* | *0.7* | *2.8* |
|  |  |  | 3 |  |  |  |  |  |
| caspase-6 inhibitor assay | positive | 45 | 8 | *m/z* 625.3, [M+H]^+^  QVEID | *98.1* | *0.9* | *0.9* | *4.9* |
|  |  |  | 5 |  |  |  |  |  |
| γ-secretase inhibition assay | positive | 71 | 0 | *m/z* 4327, [M+H]^+^  Aβ1-40 | *58.0* | *0.2* | *0.4* | *2.8* |
|  |  |  | 4 |  |  |  |  |  |
| tyrosine-kinase inhibitor assay | positive | 740 | 98 | *m/z* 616.2, [M]^+^  Heme B | *89.4* | *0.5* | *0.7* | *2.9* |
|  |  |  | 86 |  |  |  |  |  |
| CDC assay | negative | 419 | 3 | *m/z* 306.1, [M-H]^-^  GSH | *94.5* | *0.7* | *0.83* | *2.9* |
|  |  |  | 12 |  |  |  |  |  |

# Supplementary figures

#
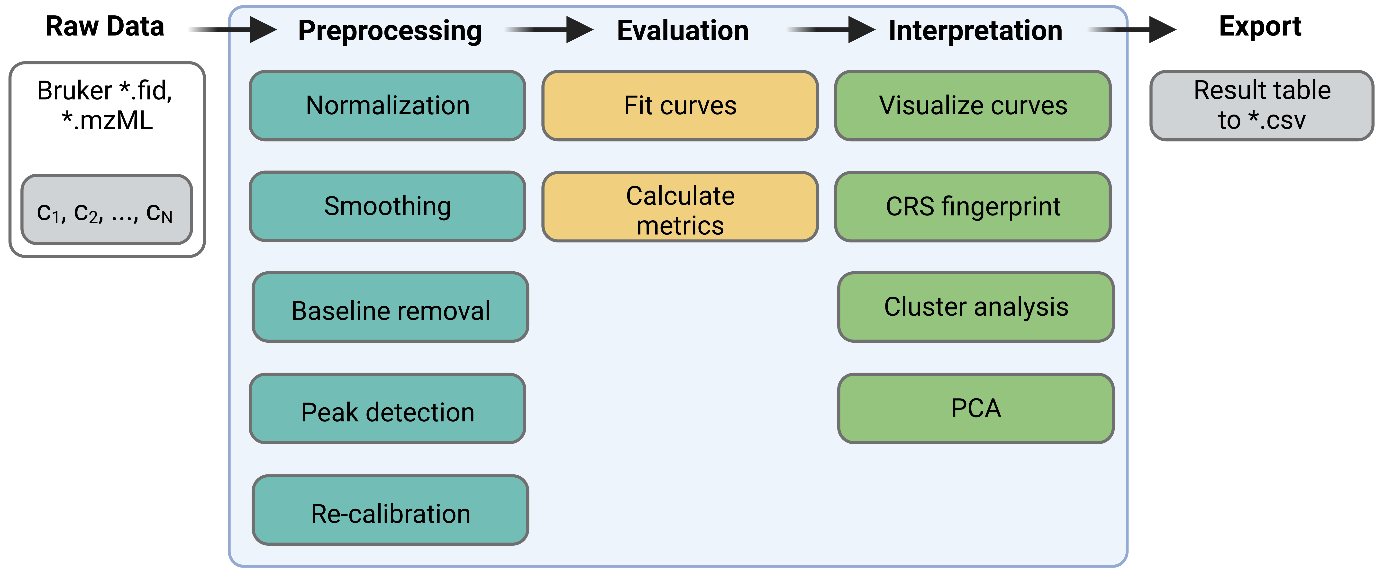


Figure S1: Schematic representation of the computational workflow of *M^2^ara*.

Raw data can be imported from Bruker *.fid files or from the open source format *.mzML. Next, data processing is performed including normalization (total ion current (TIC), median or to an internal standard), smoothing, baseline removal and peak detection. In addition, a re-calibration of the *m/z* axis can be performed using an internal standard or a known endogenous substance as reference. After preprocessing, the curves are fitted and assay quality metrics are calculated ($F_{S}$, $F_{Z}$, $F_{V}$, log_2_FC, CRS). *M^2^ara* offers different tools to interpret the data ranging from univariate (visualization of single response curves), summarizing visualizations like the CRS fingerprints enabling fast decisions, to multivariate approaches like cluster analysis and PCA. Finally, the resulting feature table can be exported to *.csv for external use.


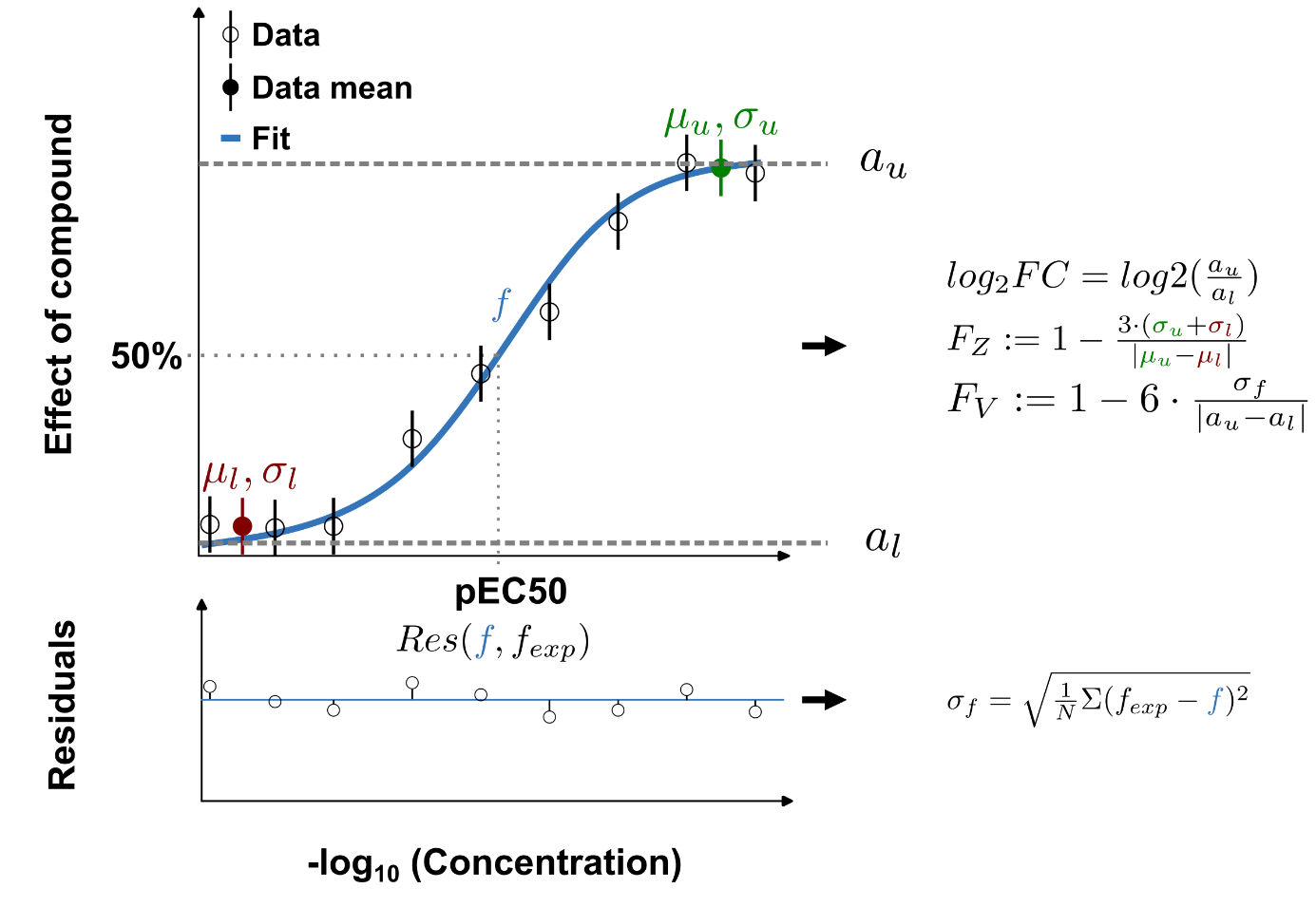


Figure S2: Graphical explanation of the used quality factors.

The $\log_{2}\mathrm{FC}$ provides the unadjusted magnitude of the curve by calculating the ratio between the upper ($a_{u}$) and lower ($a_{l}$) asymptotes. In contrast, the $F_{Z}$ factor incorporates the inherent variability of the measurements by accounting for the standard deviation ($\sigma_{u}$ and $\sigma_{l}$) of the upper ($\mu_{u}$) and lower ($\mu_{l}$) mean values, based on the values for the two highest and two lowest concentrations. To further assess the quality of the fitted curve in relation to the data, the $F_{V}$ factor is introduced, taking into account the residuals and relating them to the difference between the upper ($a_{u}$) and lower ($a_{l}$) asymptotes. Together, the proposed factors provide a holistic view of the data, allowing for differentiation between artefacts and real responses. Magnitude assesses the extent of change, variability examines consistency and noise, and model fit quality evaluates how well the data align with expected patterns—all critical for ensuring the observed changes are genuine and not artefacts.


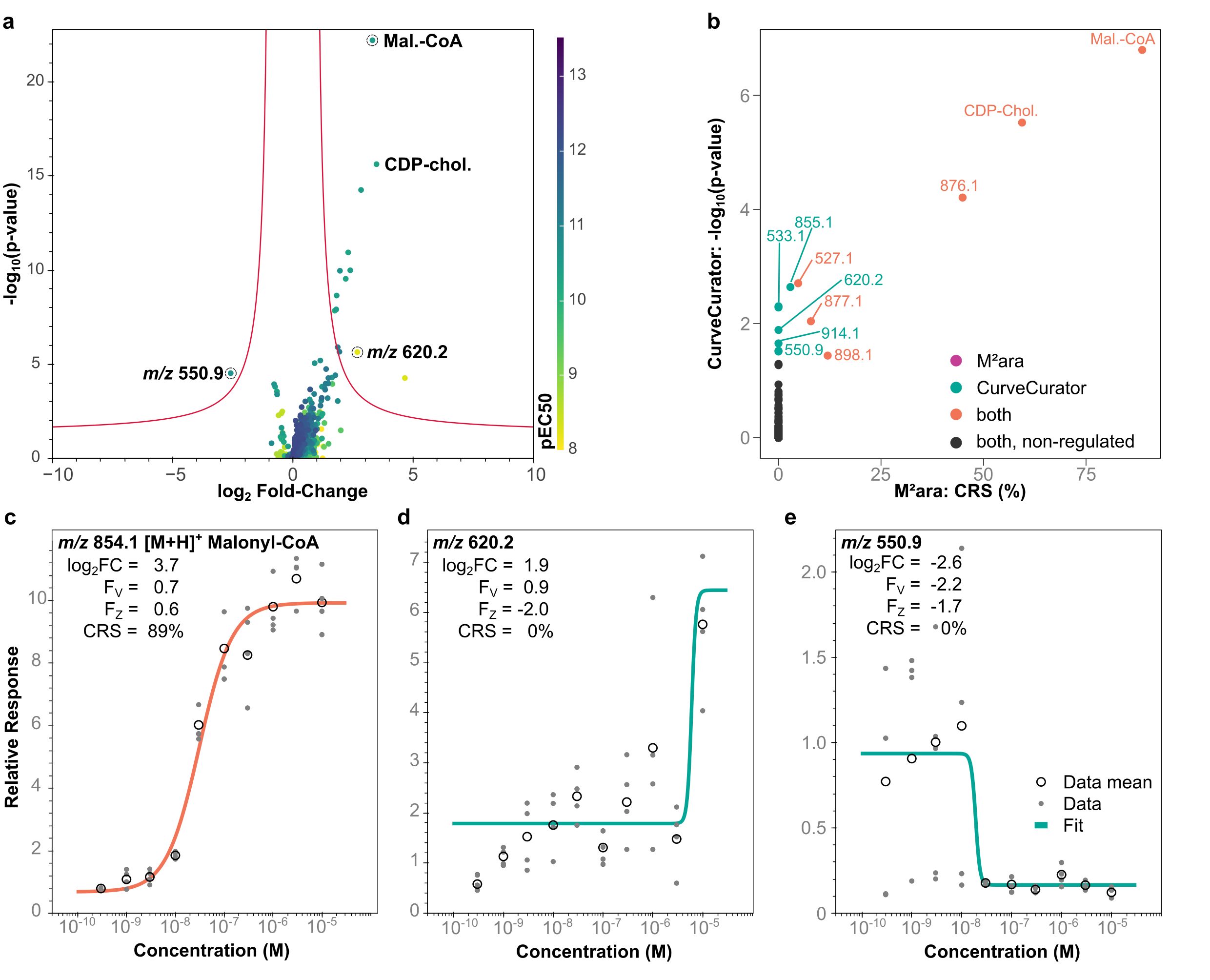


Figure S3: Results of the CurveCurator for the whole cell MALDI MS FASN assay.

**a**, Volcano plot of the significance and effect size expressed as the -log_10_(p-value) and log_2_FC, respectively. Malonyl-CoA and CDP-choline are identified as the most significant response markers. Two additional *m/z* features considered as significant using a log_2_FC threshold of 1 and a p-value threshold of 0.05 in CurveCurator are highlighted: *m/z* 550.9 and *m/z* 620.2. The color scale serves to indicate the potency that has been deduced from the fit for each feature. **b**, Direct comparison of the ranking of *m/z* features between CurveCurator (significance) and M^2^ara (curve response score). The data indicates a good agreement (red dots), with CurveCurator being more inclusive. **c-e**, Response curves determined using the CurveCurator for the three *m/z* features highlighted in **a**. Additionally, the log_2_FC, $F_{V}$, $F_{Z}$ and the CRS values deduced from M^2^ara are depicted. In M^2^ara, the two *m/z* features at *m/z* 620.2 (**d**) and *m/z* 550.9 (**e**) are considered as non-regulated based on the CRS score due to high variance in the data (**d**) as indicated by the $F_{Z}$ factor and/or a poor fit (indicated by the $F_{V}$ factor) (**e**).


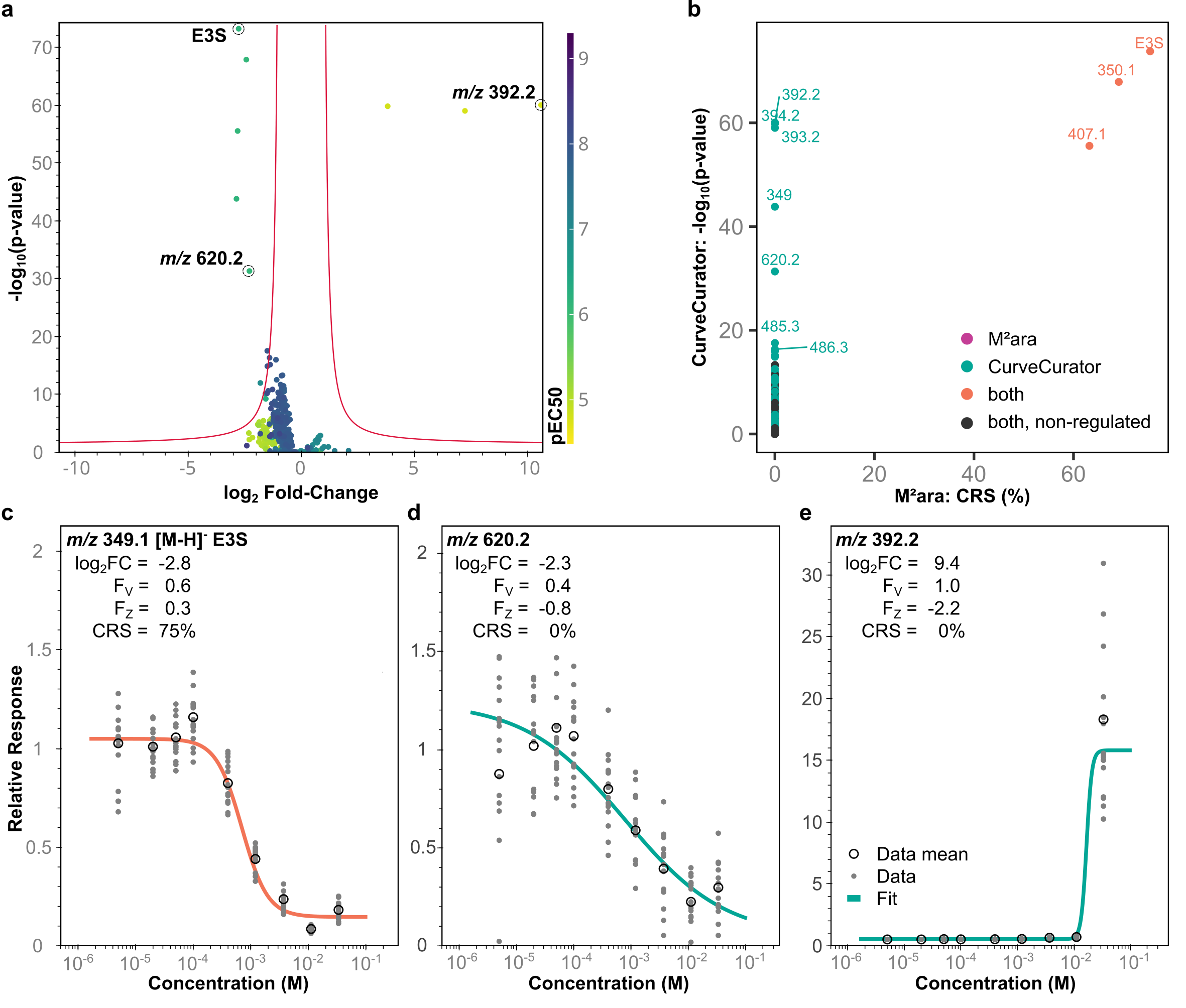


Figure S4: Results of the CurveCurator for the whole cell MALDI MS uptake and inhibition assay.

**a**, Volcano plot of the significance and effect size expressed as the -log_10_(p-value) and log_2_FC, respectively. E3S is identified as the most significant response marker. Two additional *m/z* features considered as significant using log_2_FC threshold of 1 and p-value threshold of 0.05 in CurveCurator are highlighted: *m/z* 620.2 and *m/z* 392.2. The color scale serves to indicate the potency that has been deduced from the fit for each feature. **b**, Direct comparison of the ranking of *m/z* features between CurveCurator (significance) and M^2^ara (curve response score). The data indicates a good agreement (red dots), with CurveCurator being more inclusive. **c-e**, Response curves determined using the CurveCurator for the three *m/z* features highlighted in **a**. Additionally, the log_2_FC, $F_{V}$, $F_{Z}$ and the CRS values deduced from M^2^ara are depicted. In M^2^ara, the two m/z features at *m/z* 620.2 (**d**) and *m/z* 392.2 (**e**) are considered as non-regulated based on the CRS score, due to high variance in the data (**d**) and (**e**), as indicated by the $F_{Z}$ factor.


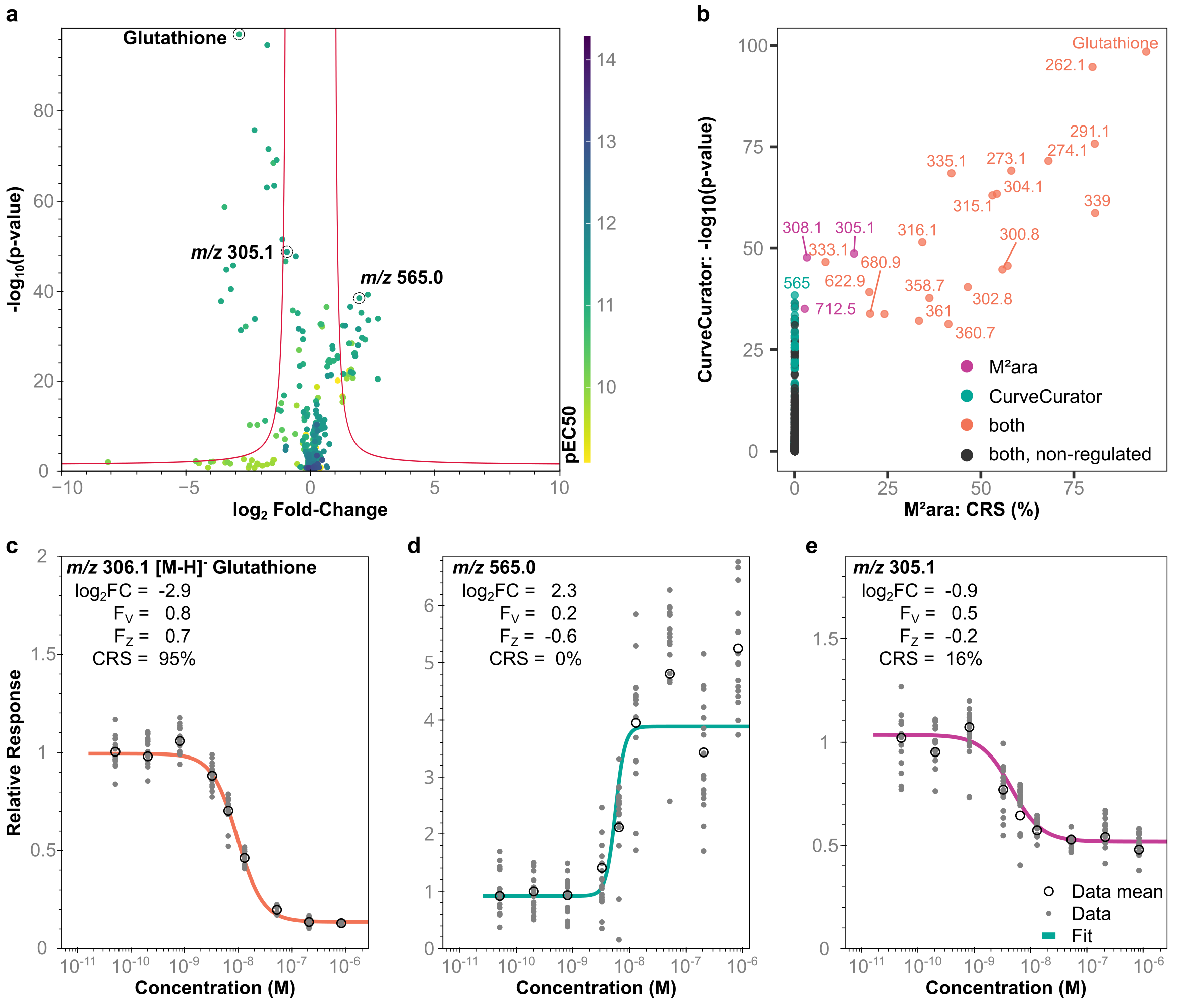


Figure S5: Results of the CurveCurator for the whole cell MALDI MS CDC assay.

**a**, Volcano plot of the significance and effect size expressed as the -log_10_(p-value) and log_2_FC, respectively. GSH is identified as the most significant response marker. Two additional *m/z* features considered as significant using log_2_FC threshold of 1 and p-value threshold of 0.05 in CurveCurator are highlighted: *m/z* 305.1 and *m/z* 565.0. The color scale serves to indicate the potency that has been deduced from the fit for each feature. **b**, Direct comparison of the ranking of m/z features between CurveCurator (significance) and M^2^ara (curve response score). The data indicates that a good agreement. (red dots) with CurveCurator being more inclusive. Some features were exclusively detected using M^2^ara (purple). **c-e**, Response curves determined using the CurveCurator for the three *m/z* features highlighted in **a**. Additionally, the log_2_FC, $F_{V}$, $F_{Z}$ and the CRS values deduced from M2ara are depicted. In M^2^ara, the *m/z* feature at m/z 565.0 (**d**) is considered as non-regulated based on the CRS score because of the high variance (indicated by the $F_{Z}$ factor). Whereas *m/z* 305.1 (**e**) would be considered as regulated by M²ara with a CRS score of 16% while CurveCurator did not consider it regulated because the log_2_FC is below. M²ara does not use a strict threshold for the log_2_FC.


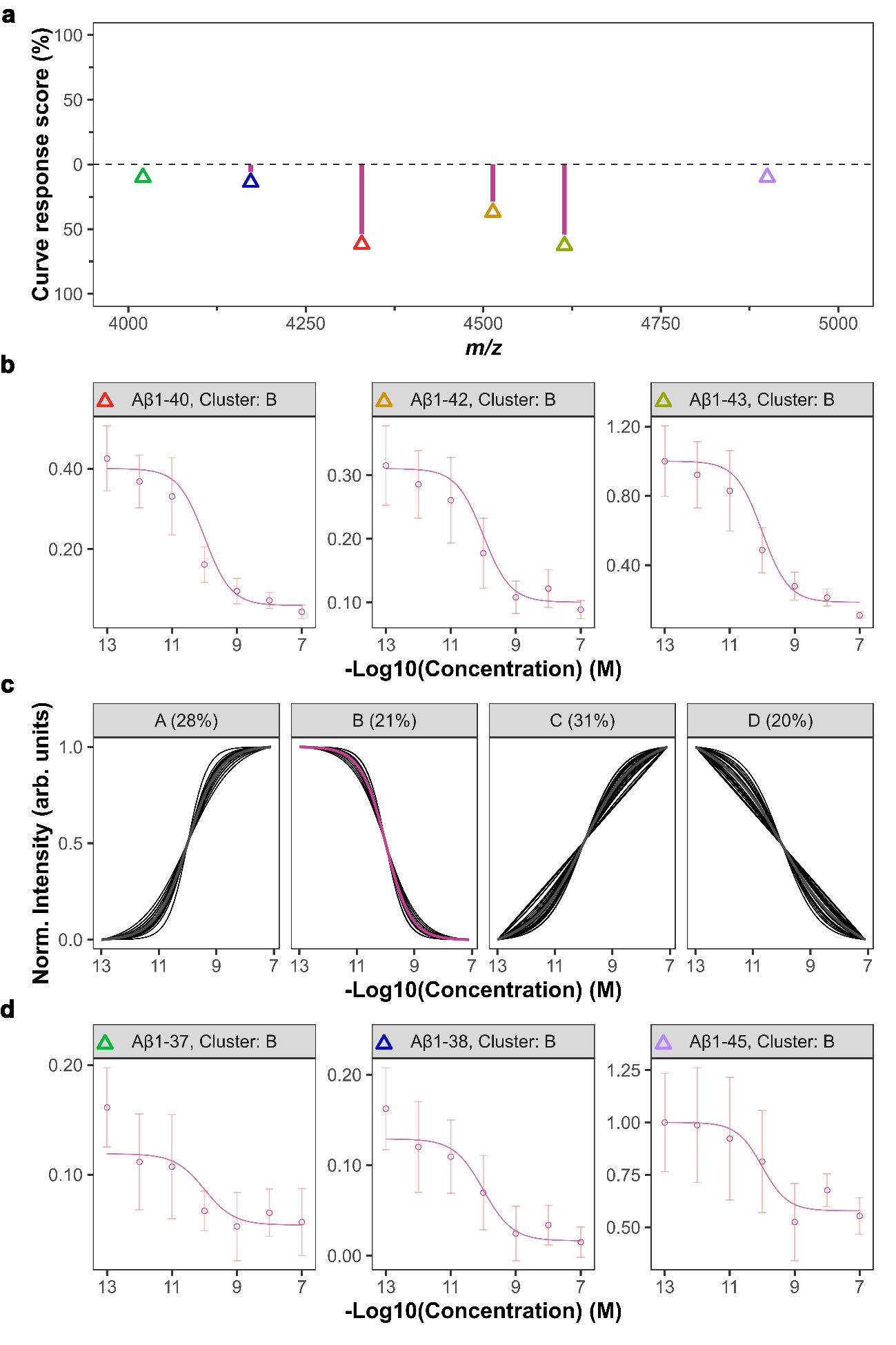


Figure S6: MALDI MS-based cell‐free γ-secretase inhibition assay of DAPT.

**a**, CRS fingerprint highlighting the down regulation of Aβ1-38 (*m/z* 4131 [M+H]^+^, blue triangle), Aβ1-40 (*m/z* 4327 [M+H]^+^, red triangle), Aβ1-42 (*m/z* 4514 [M+H]^+^, orange triangle) and Aβ1-43 (*m/z* 4615 [M+H]^+^, lawn green triangle). **b** and **d** (middle), Response curves of the aforementioned Aβ-species in the CRS fingerprint. **c**, Response curve clustering reveals that the hits form the CRS fingerprint belong to a single cluster, here cluster B. **d**, Response curves for the two known species Aβ1-37 (*m/z* 4074 [M+H]^+^, green triangle) and Aβ1-45 (*m/z* 4927 [M+H]^+^, purple triangle) that show the same general trend then aforementioned Aβ-species. Data originally published in (Koch *et al.* 2023).

**
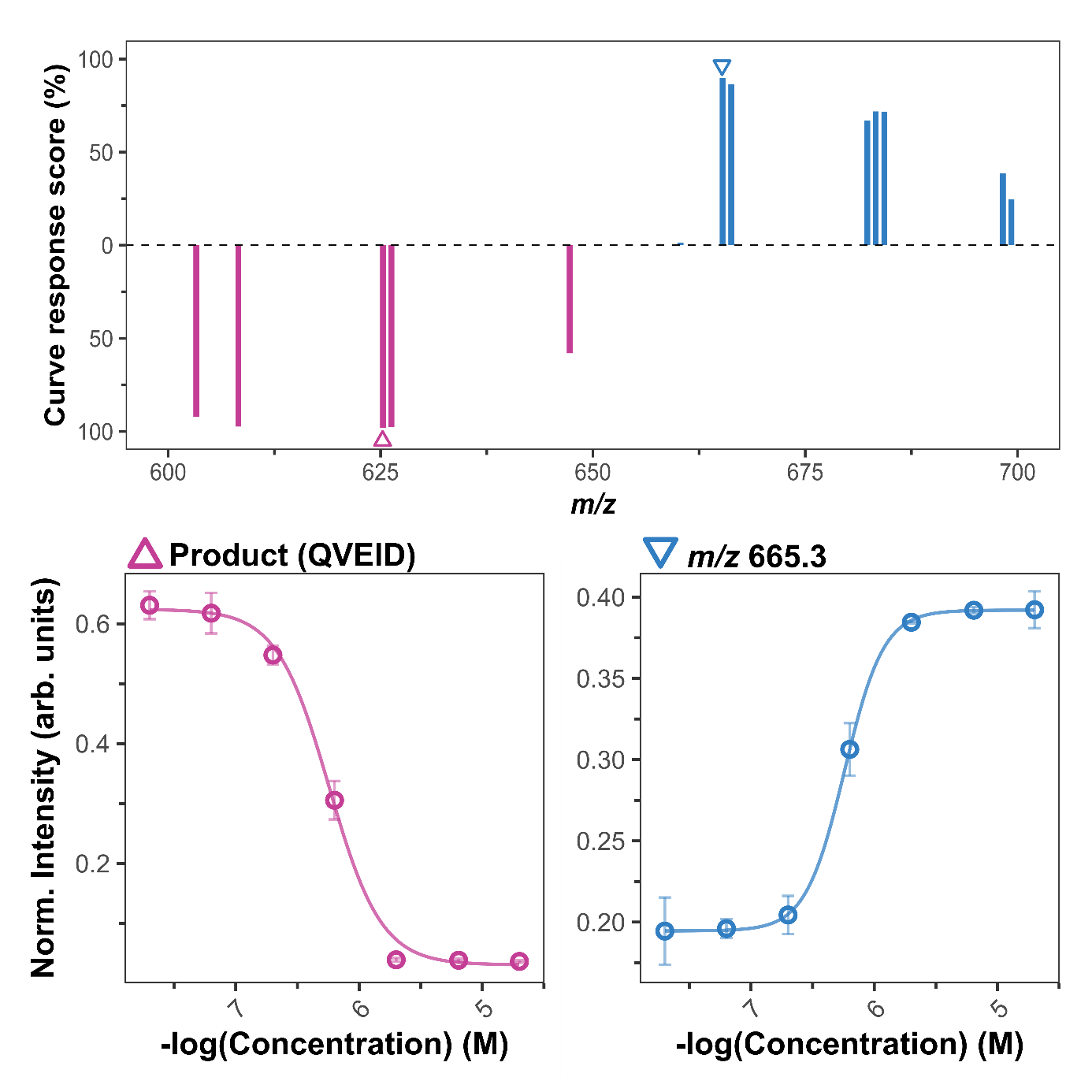
**

Figure S7: CRS fingerprint and exemplary response curves for a cell-free caspase-6 (cas-6) inhibitor assay.

Data was previously published in a white paper of Pivot Park Screening Centre ([**https://www.ppscreeningcentre.com/label-free-mass-spectrometry-ultra-highthroughput-screening/**](https://www.ppscreeningcentre.com/label-free-mass-spectrometry-ultra-highthroughput-screening/)). Caspase-6 was inhibited with Ivachtin. The upper panel shows the CRS fingerprint. Marked are the cleavage peptide product (QVEID) [M+H]^+^ at *m/z* 625.3 (purple triangle) as well as an unknown molecule with an up-regulated response curve at *m/z* 665.3 (blue triangle). The lower two panels show the response curves for the indicated molecules. In a targeted data analysis approach solely, the additional regulated features presented in the CRS fingerprint would not have been recognized.


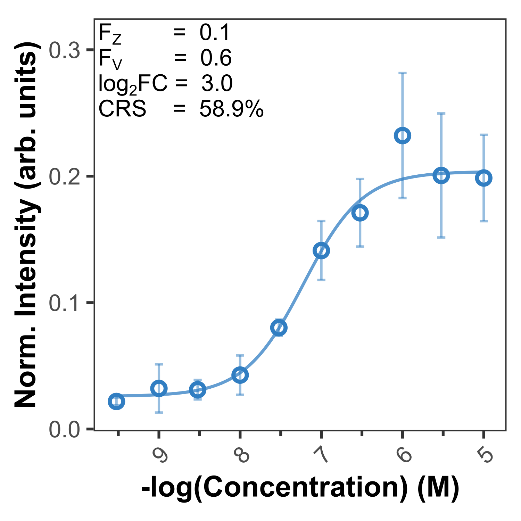


Figure S8: Characteristic response curve for *m/z* 511.1 (CDP-choline [M+H]^+^) of the FASN inhibitor assay.

As reported in Weigt et al. 2019, CDP-choline is a downstream metabolite of the Kennedy pathway that accumulates upon FASN inhibition.


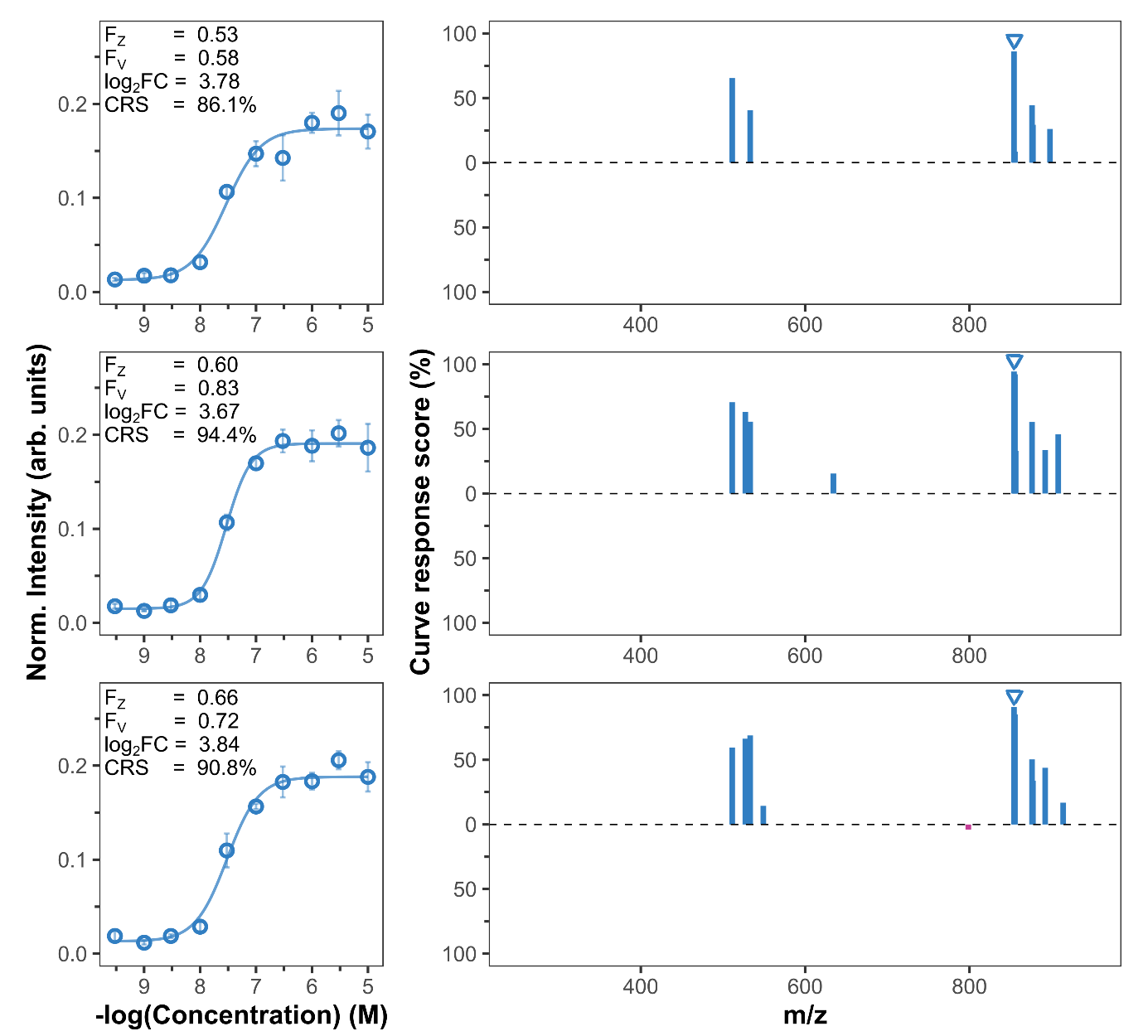


**Figure S9: Curve response analysis using *M^2^ara* of the FASN inhibitor MALDI cell assay data for three replicates.**

Left: Characteristic drug-response curves for *m/z* 854.1 (malonyl-CoA [M+H]^+^, molecular identity reported in Weigt et al. 2019) and corresponding scores. For all three replicates the $F_{Z}$ and CRS is above 0.5 and 85 %, respectively. The effect size determined by the log_2_FC is on average 3.76(8). Right: The CRS fingerprints shows similar patterns for all three replicates.


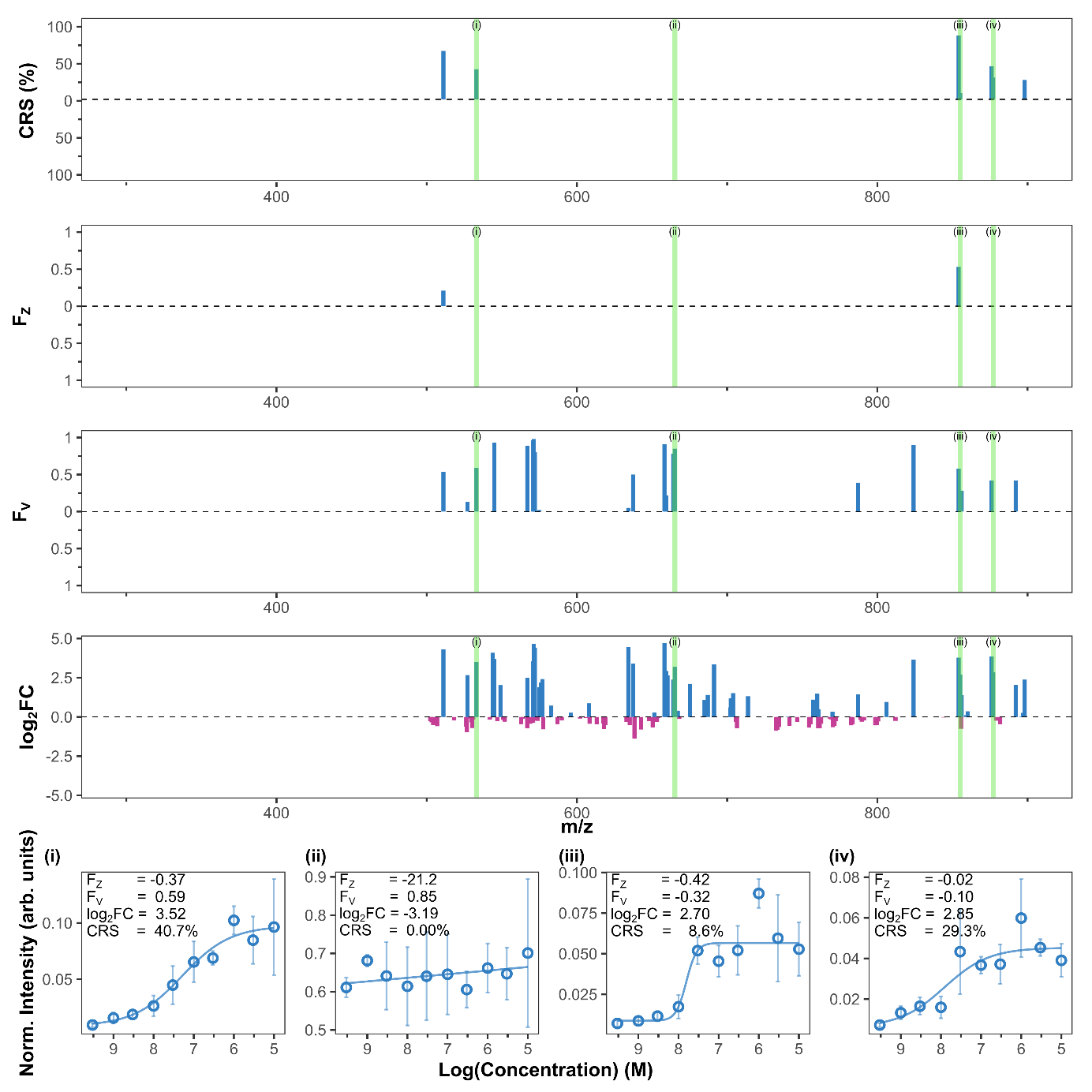


**Figure S10: Curve response fingerprints computed in *M^2^ara* of the FASN inhibitor MALDI cell assay.**

The *m/z* fingerprints for the different metrics (CRS, $F_{Z}, F_{V}$and log_2_FC) are shown from top to bottom. The lowest panel depicts the response curves of selected *m/z* features as examples on how the curve shape influences the respective scores.


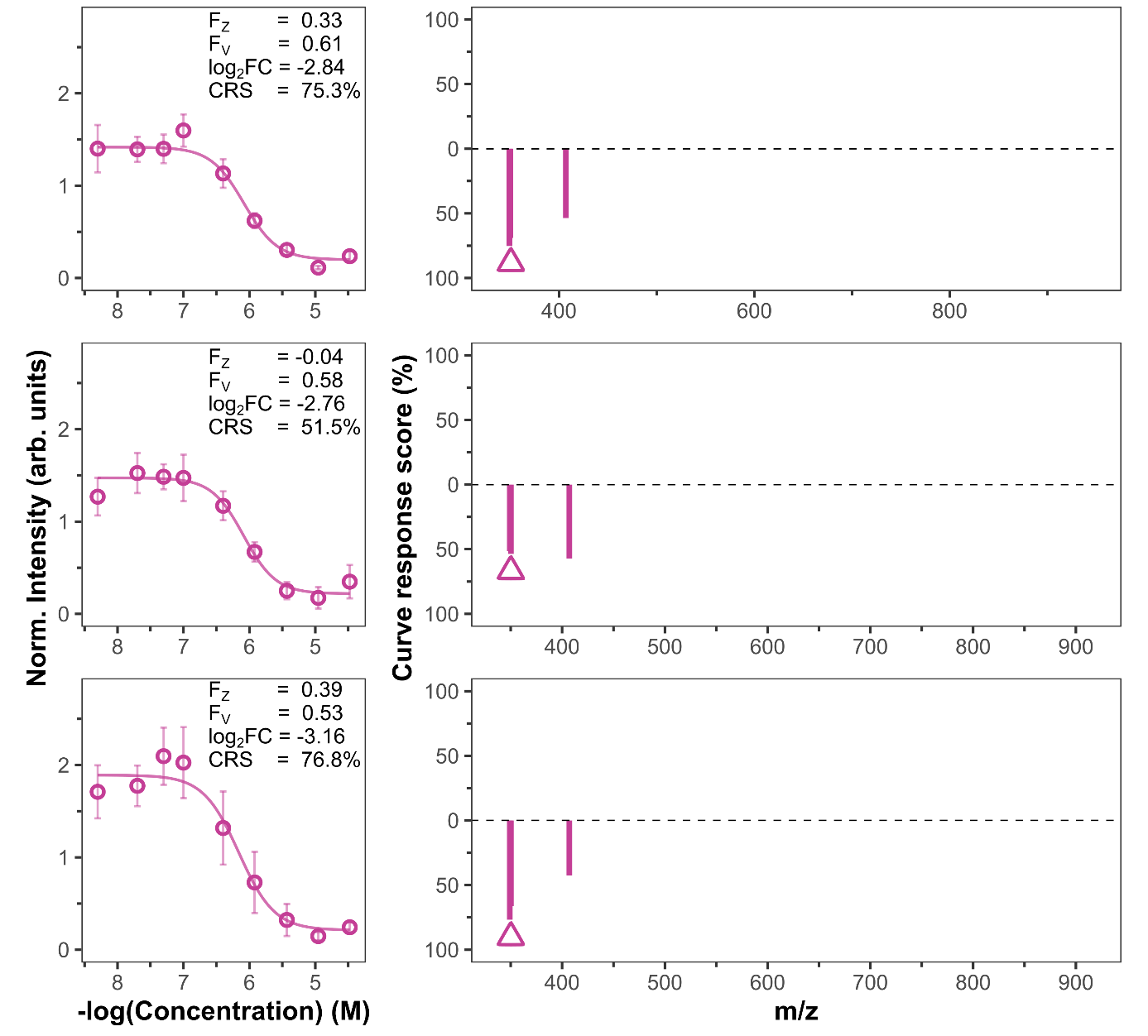


**Figure S11: Curve response analysis using *M^2^ara* of the OATP2B1 transporter MALDI cell assay for three replicates.**

Left: Characteristic drug-response curves for *m/z* 349.1 (E3S [M-H]^-^, molecular identity reported in Unger et al. 2020) and corresponding scores. For all three replicates the CRS is above 50 %. The effect size determined by the log_2_FC is on average -2.92(21). Whereas the $F_{V}$is rather similar for all replicates, the $F_{Z}$for the second replicate is reduced compared to the other two replicates. Right: The CRS fingerprints shows similar patterns for all three replicates with slight variation of the CRS for the down-regulated peaks present.

**
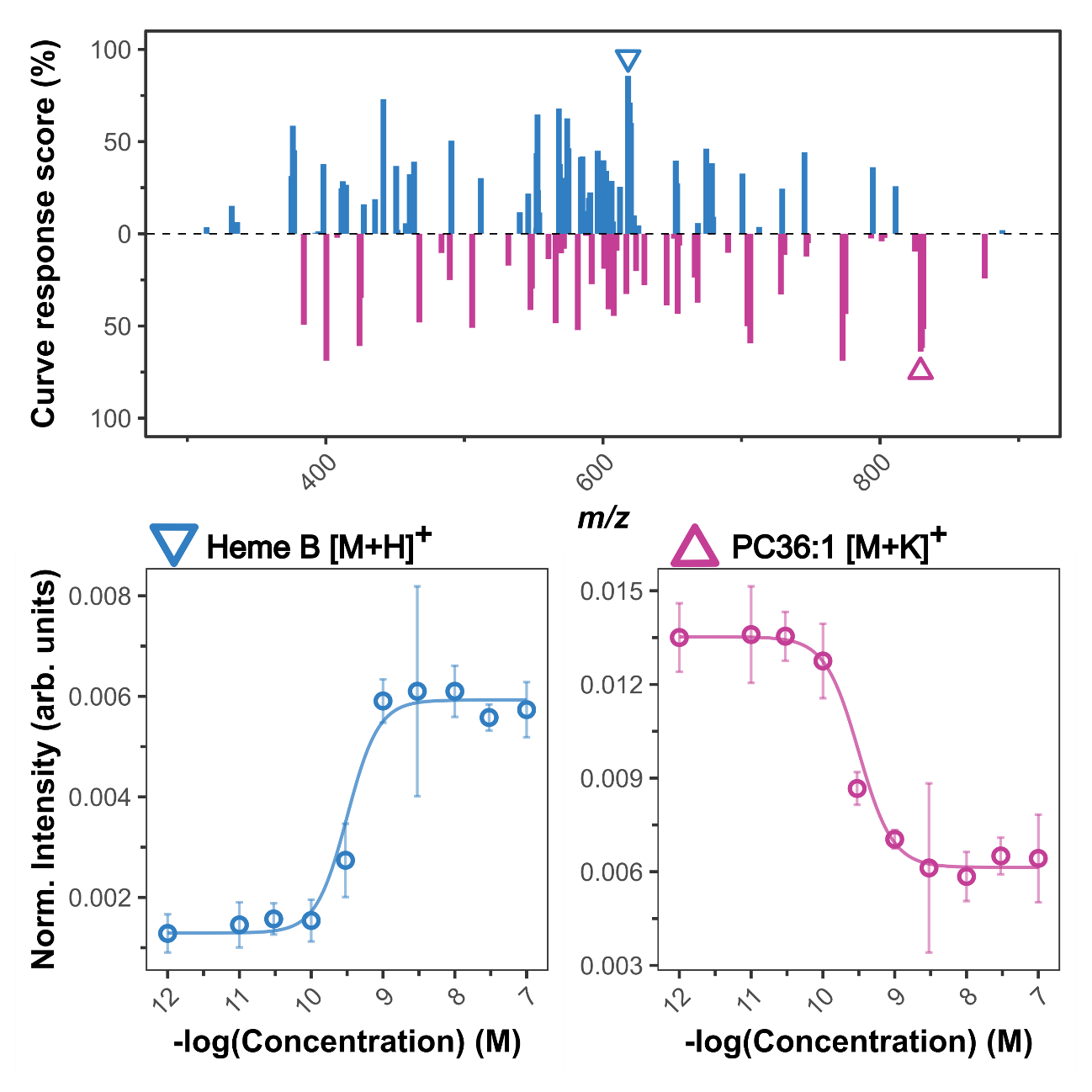
**

Figure S12: CRS fingerprint and exemplary response curves for the BCR-Abl inhibitor MALDI cell assay presented in Weigt et al., 2018.

K562 cells were treated with Dasatinib for 48h. The upper panel shows the CRS fingerprint. Marked are Heme B [M]^+^ at *m/z* 616.2 (blue triangle) as well as PC36:1 [M+K]^+^ (purple triangle). The lower two panels show the response curves for the indicated molecules.

#
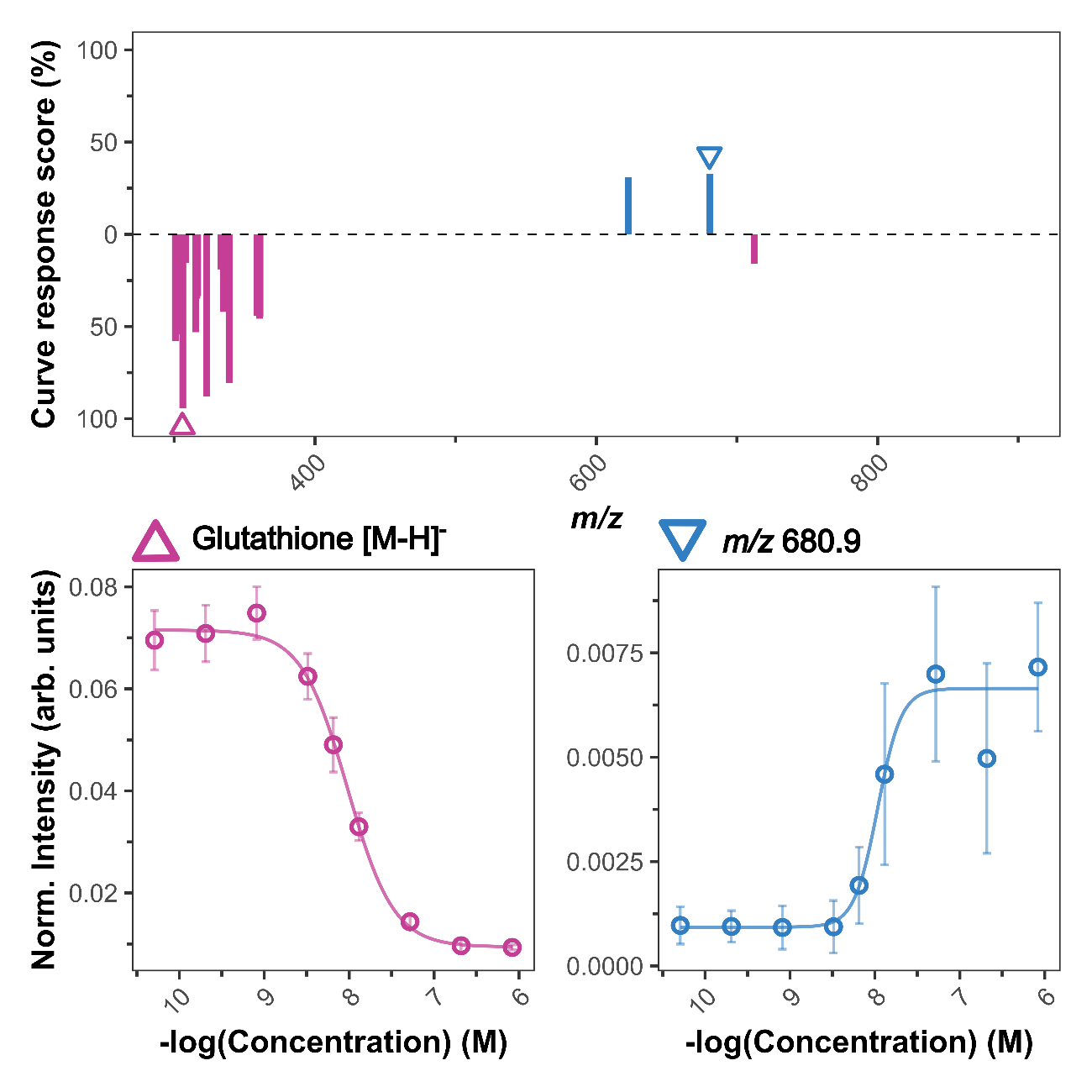


Figure S13: CRS fingerprint and exemplary response curves for the complement-dependent cytotoxicity MALDI cell assay presented in (Schmidt *et al.* 2024a).

Raji cells were treated with complement and Rituximab for 2h. The upper panel shows the CRS fingerprint. Marked are Glutathione [M-H]^-^ at *m/z* 306.1 (purple triangle) as well as *m/z* 680.9 (blue triangle). The lower two panels show the response curves for the indicated molecules.


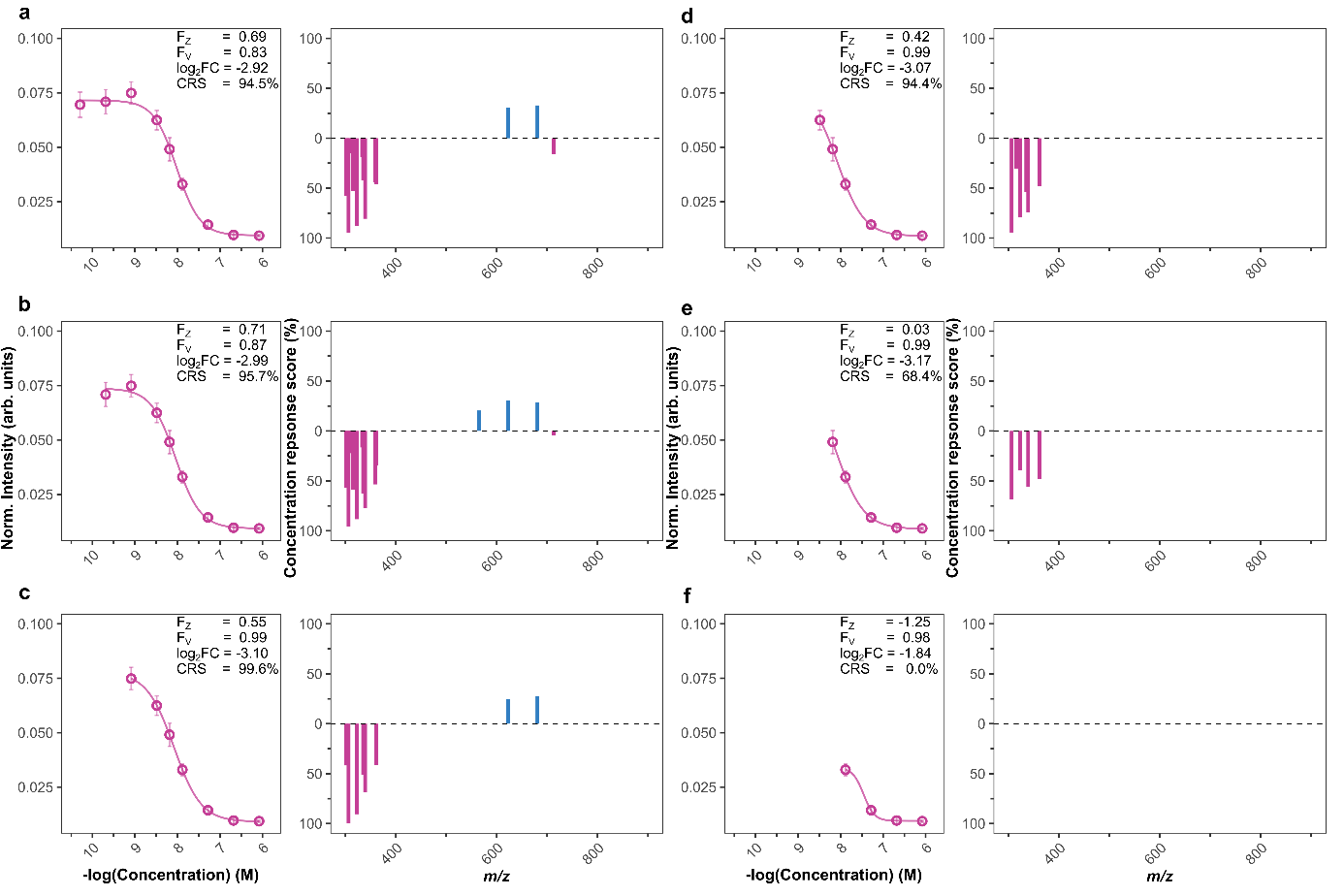


**Figure S14: Computed CRS fingerprints for demonstration the ability of *M*2*ara* to detect drug effects even if the response curve is not fully covered experimentally.**

For each condition **a-f** the data point for the lowest remaining concentration is removed from the data set. Corresponding curve fits, scores and CRS fingerprints are depicted. Data taken from(Schmidt *et al.* 2024b). Curves correspond to Glutathione [M-H]^-^ at *m/z* 306.1.


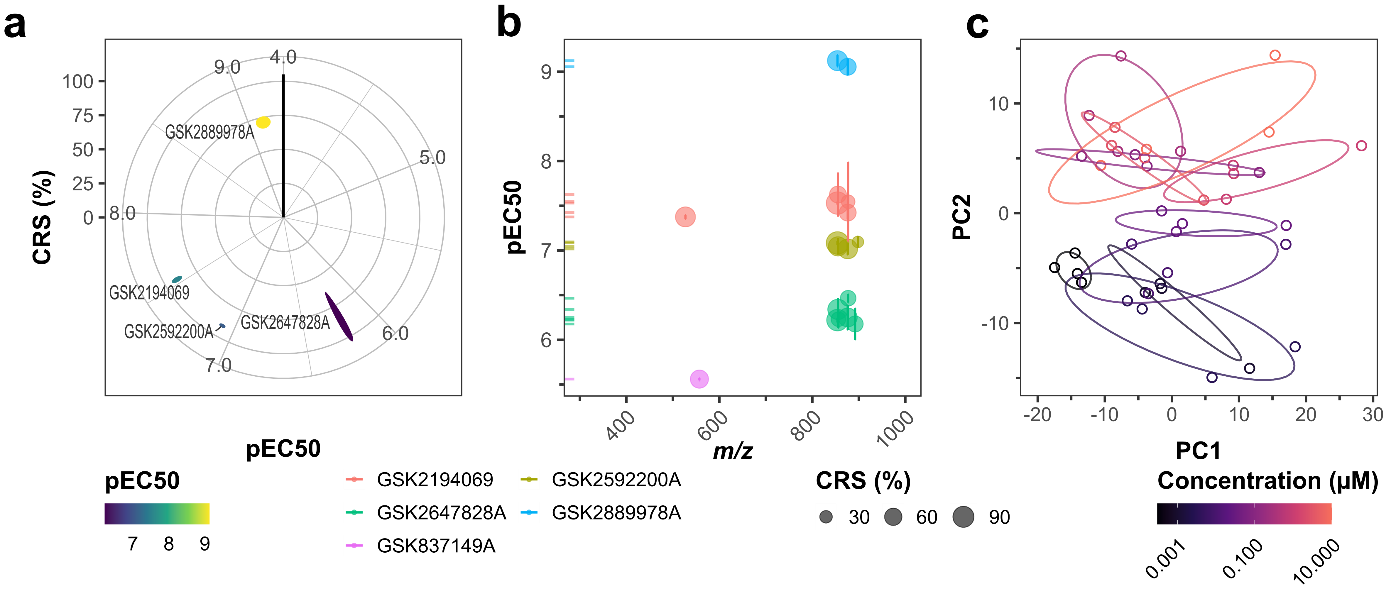


Figure S15: Characteristics of FASN inhibitors.

**a**, Polar plot of the CRS and the pEC50 value of different drugs used in Weigt, et. al. 2019. Uncertainties in the pEC50 and CRS dimension are given by the size of the ellipses. **b**, pEC50 for responding *m/z* features for different drugs used in Weigt, et. al. 2019 highlighting the potential of *M^2^ara* to reveal complex cellular response patterns for different treatments. **c**, PCA of MALDI spectra form Weigt, et. al. 2019 highlighting the separation in high dimensional space by different concentrations of GSK2194069. Note that the data was processed with *M²ara* individually but externally united and visualized.

# References

Bray M-A, Carpenter A. Advanced Assay Development Guidelines for Image-Based High Content Screening and Analysis. In: Markossian S, Grossman A, Arkin M, et al. (eds.). *Assay Guidance Manual*. Bethesda (MD): Eli Lilly & Company and the National Center for Advancing Translational Sciences, 2004.

Commo F, Bot BM. nplr: N-Parameter Logistic Regression. 2016.

Giraldo J, Vivas NM, Vila E *et al.* Assessing the (a)symmetry of concentration-effect curves: empirical versus mechanistic models. *Pharmacol Ther* 2002;**95**:21–45.

Iversen PW, Eastwood BJ, Sittampalam GS *et al.* A comparison of assay performance measures in screening assays: signal window, Z’ factor, and assay variability ratio. *J Biomol Screen* 2006;**11**:247–52.

Koch M, Enzlein T, Chen S-Y *et al.* APP substrate ectodomain defines amyloid-β peptide length by restraining γ-secretase processivity and facilitating product release. *The EMBO Journal* 2023;**n/a**:e114372.

Ravkin I, Temov V, Nelson AD *et al.* Multiplexed high-throughput image cytometry using encoded carriers. *Imaging, Manipulation, and Analysis of Biomolecules, Cells, and Tissues II*. Vol 5322. SPIE, 2004, 52–63.

Schmidt S, Geisel A, Enzlein T *et al.* Label-free assessment of complement-dependent cytotoxicity of therapeutic antibodies via a whole-cell MALDI mass spectrometry bioassay. *Sci Rep* 2024b;**14**:21462.

Zhang JH, Chung TD, Oldenburg KR. A Simple Statistical Parameter for Use in Evaluation and Validation of High Throughput Screening Assays. *J Biomol Screen* 1999;**4**:67–73.

Zhang XD. A pair of new statistical parameters for quality control in RNA interference high-throughput screening assays. *Genomics* 2007;**89**:552–61.
